# *In Silico* Analysis of **β**-Galactosidases Primary and Secondary Structure in relation to Temperature Adaptation

**DOI:** 10.1155/2014/475839

**Published:** 2014-03-24

**Authors:** Vijay Kumar, Nikhil Sharma, Tek Chand Bhalla

**Affiliations:** Department of Biotechnology, Himachal Pradesh University, Summer Hill, Shimla, Himachal Pradesh 171005, India

## Abstract

**β**-D-Galactosidases (EC 3.2.1.23) hydrolyze the terminal nonreducing **β**-D-galactose residues in **β**-D-galactosides and are ubiquitously present in all life forms including extremophiles. Eighteen microbial **β**-galactosidase protein sequences, six each from psychrophilic, mesophilic, and thermophilic microbes, were analyzed. Primary structure reveals alanine, glycine, serine, and arginine to be higher in psychrophilic **β**-galactosidases whereas valine, glutamine, glutamic acid, phenylalanine, threonine, and tyrosine are found to be statistically preferred by thermophilic **β**-galactosidases. Cold active **β**-galactosidase has a strong preference towards tiny and small amino acids, whereas high temperature inhabitants had higher content of basic and aromatic amino acids. Thermophilic **β**-galactosidases have higher percentage of **α**-helix region responsible for temperature tolerance while cold loving **β**-galactosidases had higher percentage of sheet and coil region. Secondary structure analysis revealed that charged and aromatic amino acids were significant for sheet region of thermophiles. Alanine was found to be significant and high in the helix region of psychrophiles and valine counters in thermophilic **β**-galactosidase. Coil region of cold active **β**-galactosidase has higher content of tiny amino acids which explains their high catalytic efficiency over their counterparts from thermal habitat. The present study has revealed the preference or prevalence of certain amino acids in primary and secondary structure of psychrophilic, mesophilic, and thermophilic **β**-galactosidase.

## 1. Introduction

Microbes are widespread in most diverse environmental conditions including extreme salinity, pressure, pH, and temperature. These microbes, called extremophiles, produce enzymes which are capable of working under extreme conditions and attract much attention due to their industrial importance [1] and basic interest of science. Proteins undergo denaturation at both extreme ends of temperature, cold denaturation due to the temperature dependence of the hydrophobic effect [2] and thermal denaturation at high temperatures. The emphasis of a cold active protein is more on function than on structure and there has been much interest in thermophiles due to the possibility that life has a thermophilic origin, in deep-sea vents [3], and also due to their important biotechnological applications at higher temperatures.

**β**-Galactosidases (EC 3.2.1.23) are among the most diverse enzymes on earth found in almost all life forms inhabiting at near zero to near 100 temperature. This enzyme is known to catalyze both hydrolytic cleavage of **β** (1–4) linkage of lactose to glucose and galactose [4] and transglycosylation reactions, that is, transfer the galactose formed from lactose cleavage onto the galactose moiety of another lactose to yield galactooligosaccharides [5, 6]. This enzyme is present in psychrophiles [4, 7], mesophiles [8], thermophiles [9, 10], and hyperthermophiles [11, 12]. Cold active *β*-galactosidases present in cold-adapted organisms thriving in earth's polar regions and other areas, where the mean annual temperature is below 5°C, and thermoactive and thermostable counterpart occurs in microbes inhabiting thermal springs. *β*-Galactosidases from extremophiles are of much interest because of their suitability in various industrial processes. Cold active *β*-galactosidase that hydrolyzes lactose is of high biotechnological interest, particularly for removal of lactose in milk and dairy products at low temperatures and ethanol production from wheat. Due to wide distribution in varying temperature conditions and their industrial applications, *β*-galactosidases are one of the best model proteins to study the relationship between amino acid composition at primary and secondary structure in relation to their temperature adaptation. Therefore the present investigation has been focused to find out the crucial amino acids residues and their percentage composition in primary and secondary structure of *β*-galactosidases from the well characterized microbes from psychrophilic, mesophilic, and thermophilic microbes.

## 2. Material and Methods 

### 2.1. Sequence Retrieval

Nucleotide and protein sequences of *β*-galactosidase of thermophiles, mesophiles, and psychrophiles were retrieved from NCBI (http://www.ncbi.nlm.nih.gov/) and UniProt proteomic server (http://www.uniprot.org/), respectively. Information about the optimum temperature of some microbial *β*-galactosidases was obtained from BRENDA (http://www.brenda-enzymes.org/) and the experimental data earlier published (Table 1).

### 2.2. Calculation of Physiochemical Properties

Physiochemical data were generated from the SwissProt and Expert Protein Analysis System (ExPASy) (http://www.expasy.org/), that is, the proteomic server of the Swiss Institute of Bioinformatics (SIB). Blastp (Protein BLAST) (http://blast.ncbi.nlm.nih.gov/) was performed to study the homology among the various *β*-galactosidase sequences and six sequences each belonging to thermophiles, mesophiles, and psychrophiles were selected (Table 1).

### 2.3. Composition of Amino Acids at Primary Structure and Secondary Structure

Protparam tools (http://web.expasy.org/protparam/) in the ExPASy proteomic server were applied to calculate/deduce percentage of individual amino acids of *β*-galactosidase from the primary protein sequences. Percentages of amino acids according to their properties were calculated by using Pepstats at Mobyle portal (http://mobyle.pasteur.fr/cgi-bin/portal.py#forms::pepstats/al.). Secondary structure was predicted by Psipred (http://bioinf.cs.ucl.ac.uk/psipred/) and percentage composition was calculated by Protparam.

### 2.4. Statistical Analysis

An analysis of variance (ANOVA) was conducted on various physiochemical parameters and composition of individual amino acids at primary structure and secondary structures for each study with the statistical package “*Assistat version-7.6 beta 2013.*”* F*-tests were used to determine the statistical significance. Tukey's test was applied for all pairwise comparisons of mean responses.

## 3. Results

Eighteen microbial *β*-galactosidase nucleotide and protein sequences were retrieved from NCBI and UniProt protein databank, respectively, having well characterized data regarding their temperature requirement (Table 1). Dataset was divided into three groups, that is, thermophiles, mesophiles, and psychrophiles, according to their optimum temperature for *β*-galactosidase activity. Different physiochemical properties were predicted using ExPASy server, that is, length of amino acid chain, pI, molecular weight, extinction coefficient, instability index, aliphatic index, GRAVY, and so forth. None of these physiochemical properties was found to be statistically significant with regard to temperature adaptation.

### 3.1. Amino Acid Distribution at Primary Structure of Different *β*-Galactosidases

**Figure 1:**
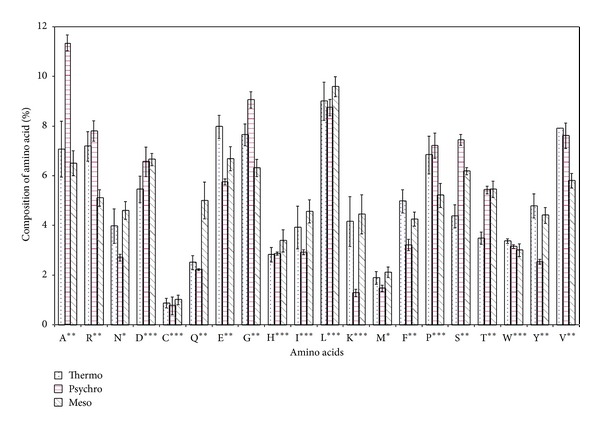
Percentage composition of amino acid in *β*-galactosidase from some thermophilic, psychrophilic, and mesophilic microorganisms. Error bar represents standard error (SE = SD/$\sqrt{n}$, where *n* is number of sample and SD is standard deviation). **Significative at a level of 1% of probability (*P* < 0.01). *Significative at a level of 5% of probability (0.01 ≤ *P* < 0.05). ***Nonsignificative (*P* ≥ 0.05).

Analysis of percentage composition of individual amino acids of *β*-galactosidase at primary structure (Figure 1) revealed that the roles of alanine, valine, arginine, glutamine, glutamic acid, glycine, phenylalanine, serine, threonine, and tyrosine in temperature adaptation were most significant. Asparagine and methionine were also important from temperature adaptation point of view. Glutamic acid, cysteine, isoleucine, leucine, histidine, proline, and tryptophan were statistically nonsignificant. Alanine content was very high in psychrophilic *β*-galactosidase as compared to its thermophilic and mesophilic counterparts. Arginine, glycine, and serine were marginally higher in cold loving *β*-galactosidase, whereas thermophilic *β*-galactosidase had slightly high content of glutamic acid, phenylalanine, tyrosine, and valine. Asparagine content was very low as we move towards extreme temperature from moderate. Cysteine and methionine which were supposed to be key residue for temperature adaptation due to their capability to form disulfide bond were found almost equal in this study.

On a major division of amino acids, tiny, small, basic, and aromatic amino acids were turned out to be most significant. Psychrophilic *β*-galactosidase had higher amount of tiny and small amino acids as compared to thermophilic and mesophilic *β*-galactosidases which had almost similar amount of these amino acids. Aromatic and basic amino acid increased in percentage as we go from psychrophiles to thermophiles. A percentage of polar amino acids were higher while nonpolar were lesser in mesophilic *β*-galactosidase as compared to psychrophilic and thermophilic *β*-galactosidases (Figure 1).

### 3.2. Amino Acid Distribution at Secondary Structure of Different *β*-Galactosidases

Secondary structure of the above-mentioned *β*-galactosidase from some thermophilic, psychrophilic, and mesophilic microorganisms was predicted. Percentage count of *α*-helix, *β*-pleated sheet, and coil region were calculated. Statistically the percent count of various amino acids which are responsible for the formation of helix, sheet, and coil region was nonsignificant when individually calculated for the given regions (Figure 3). However, thermophilic *β*-galactosidase had marginally higher percentage of *α*-helix whereas cold loving *β*-galactosidase had higher percentage of sheet and coil region as compared to its other counterparts.

#### 3.2.1. Amino Acid Composition in Coil Region

Analysis of amino acid composition in coil region of *β*-galactosidases from some microbes having varied temperature range showed three amino acids, that is, alanine, glycine, and phenylalanine, to be statistically significant. Alanine content had almost twofold increase of content whereas phenylglycine was low in psychrophilic *β*-galactosidase as compared to thermophilic *β*-galactosidases. Percentage of glycine was higher at both extreme ends as compared to moderate temperature. Arginine and proline were slightly higher in *β*-galactosidase of psychrophiles and thermophiles whereas mesophiles have marginally higher percentage of asparagine and glutamic acid. Polar amino acids, that is, serine and tyrosine, were low in coil region of *β*-galactosidase of thermophiles (Figure 4).

#### 3.2.2. Amino Acid Composition in Helix Region

Alanine content was exceptionally higher in helix region of *β*-galactosidase of psychrophiles while valine was higher in thermophiles. Lysine was present at very low percentage in extremophiles. Glutamine was present at a very small amount in thermophilic *β*-galactosidase, whereas its content was comparatively higher in psychrophilic and in mesophilic *β*-galactosidase. Aspartic acid was threefold higher in psychrophilic *β*-galactosidase as compared to mesophilic and thermophilic *β*-galactosidase. Tyrosine, another statistically significant amino acid besides the above-mentioned, was increased in amount as the optimum temperature of *β*-galactosidase rose.

Glutamic acid, phenylalanine, and tryptophan were statistically less significant in helix region of *β*-galactosidases. Glutamic acid showed similar trend as aspartic acid, whereas aromatic amino acid phenylalanine was high in thermophilic *β*-galactosidases. Tryptophan was almost similar in *β*-galactosidase of extremophiles and slightly lesser as compared to mesophiles. The rest of the amino acids were statistically insignificant. Glycine was higher in thermophiles followed by psychrophiles and mesophiles. Proline, the other amino acid which disturbs the helix structure, was higher in psychrophiles when compared to mesophilic and thermophilic *β*-galactosidases (Figure 5).

#### 3.2.3. Amino Acid Composition in Sheet Region

Sheet regions of *β*-galactosidase from different temperature inhabitants have seven statistically significant amino acids, that is, glutamine, glutamic acid, glycine, serine, threonine, tyrosine, and valine. Besides these amino acids, lysine, histidine, and aspartic acid were the other statistically less significant amino acids. Positively charged amino acids glutamic acid and aspartic acid and negatively charged amino acid lysine were higher in thermophilic *β*-galactosidase. Polar amino acids serine, threonine, and tyrosine all were statistically significant in sheet region of *β*-galactosidase. Serine and threonine were low in percentage in thermophilic *β*-galactosidase and aromatic amino acid tyrosine was high in thermophiles whereas the reverse is for psychrophilic *β*-galactosidase. Valine and glycine were present in decreasing order as we go high towards higher temperature. Psychrophilic *β*-galactosidase had lower content of asparagine and glutamine as compared to its relative from higher temperature (Figure 6).

## 4. Discussion

The exact role of amino acids in protein temperature adaptation has been in long studies which have shown the involvement of amino acids in temperature adaptation of proteins and we stretch these findings being contributory to thermoadaptive properties of *β*-galactosidase. In psychrophiles individual residue compositions show that there are a significant preference for A (Ala), R (Arg), G (Gly), S (Ser), and T (Thr) content and significant avoidance of E (Glu), F (Phe), and Y (Tyr) content and moderate preference for D (Asp) and P (Pro) and avoidance of I (Ile), L (Leu), and K (Lys) content (Table 1). All these residue preferences and avoidance directly show a strong correlation with respect to avoidance for helical content in psychrophiles, as S (Ser), D (Asp), and G (Gly) are helix breakers [28] and T (Thr) is helix indifferent. This trend is reverse in thermophilic *β*-galactosidase, as there is abundance of amino acid favoring helix. Amino acid D (Asp) is observed to be unstable at high temperatures and therefore its frequency is observed to decrease as optimal growth temperature of organisms increases [29]. Reverse trends are observed for E (Glu) to counter the trend in favor of making ion pair interactions to form salt bridges at higher temperatures [30, 31]. Moreover, thermolabile amino acid residues like asparagine, glutamine, and methionine should be less in thermostable *β*-galactosidase as they tend to undergo oxidation and deamination at elevated temperatures [32, 33]. This is also supported by our result which shows lesser percentage of thermolabile amino acid residues in thermostable *β*-galactosidase (Figure 1). Helixes destabilizing *β*-branched residues (T (Thr) and V (Val)) are preferred in beta sheets and loop regions of psychrophilic proteins [34, 35]. Amino acids with aliphatic, basic, aromatic, and hydrophilic side chains are underrepresented in the helical regions of proteins of psychrophiles [36]. This again supports the amino acid distribution data presented here (Figures 4–6). Cold active enzymes display a high catalytic efficiency and compromised thermal stability. In most cases, the adaptation to cold is achieved through a reduction in the activation energy that possibly originates from an increased flexibility of either a selected area or the overall protein structure. This enhanced plasticity seems in turn to be induced by the weak thermal stability of psychrophilic enzymes [37]. On the other hand high temperature inhabitants have more compact structure, tight helix, higher interaction in form of salt bridge, and hydrogen bond among its amino acid residues. The composition of amino acid of psychrophilic, mesophilic, and thermophilic *β*-galactosidase in three major secondary structural elements, *α*-helices, *β*-sheets, and coils, is given in Figure 3. Collectively taken, the psychrophilic and mesophilic *β*-galactosidase contain a significantly smaller number of residues (14% and 15%, resp.) in the *α*-helices as compared to thermophiles (20%). On contrary to this sheet region of thermophilic protein have lower percentage of residues (26%) as compared to mesophilic (29%) and psychrophilic *β*-galactosidase (32%). This showed that more helix regions give the protein advantage at higher temperature while sheet region was thermolabile and thereby present in low amount in thermophilic protein. A similar kind of trend was observed in proteome analysis of some psychrophilic and mesophilic bacterial species [37]. Coil region is however almost comparable in the present dataset.

### 4.1. Charged Amino Acids

**Figure 2:**
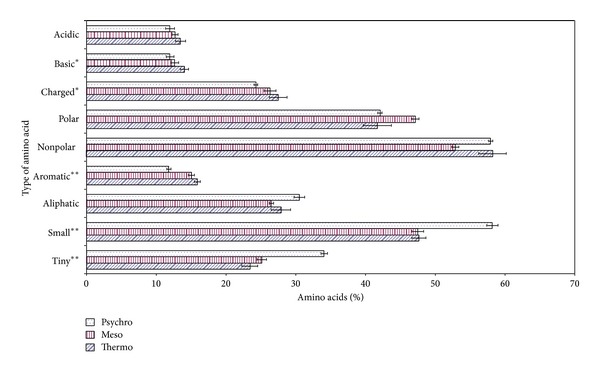
Percentage composition of amino acid in *β*-galactosidases from some thermophilic, psychrophilic, and mesophilic microorganisms.

**Figure 3:**
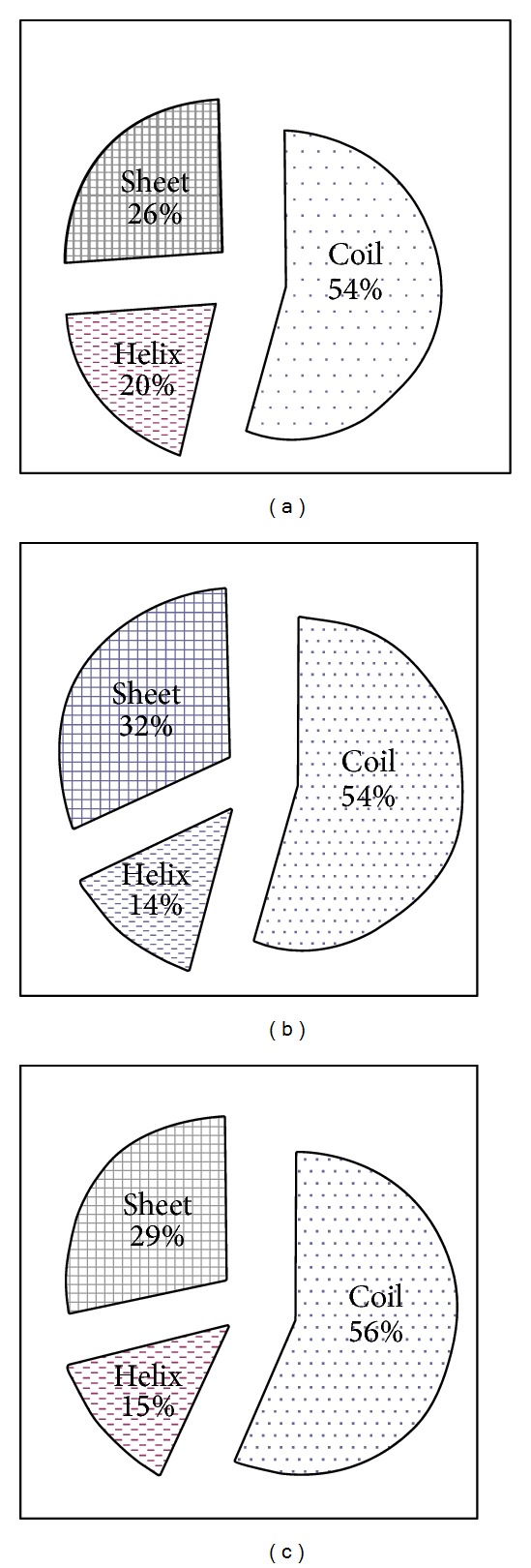
Percentage composition of coil, helix, and sheet region in *β*-galactosidase from (a) thermophilic, (b) psychrophilic, and (c) mesophilic microorganisms.

**Figure 4:**
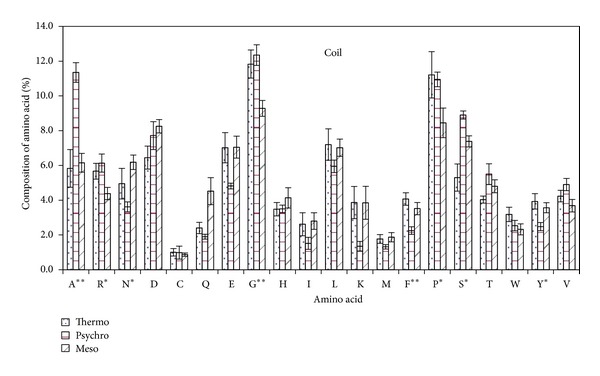
Percentage composition of amino acid in coil region of *β*-galactosidase from some thermophilic, psychrophilic, and mesophilic microorganisms.

It had been shown that the presence of more charged residues leads to thermostability of proteins as they are involved in electrostatic interactions which stabilize the secondary structure of protein [32, 38] and form more salt bridges [39]. The higher content of amino acids involved in long-range interactions has been proposed as a mechanism for maintaining conformation at high temperatures [40]. The present data also confirmed the presence of more charged residues in thermophilic *β*-galactosidase than mesostable and psychrophilic ones (Figure 2). However, only glutamic acid was present in higher amount in thermophilic *β*-galactosidase and other charged amino acids; that is, aspartic acid, arginine, lysine, and histidine were comparable at primary structure of other groups of *β*-galactosidase (Figure 1), but the actual role of charged amino acids in relation to temperature adaptation lies in sheet region. In sheet region of the present dataset, the charged amino acids, that is, aspartic acid, glutamic acid, and lysine, were present in higher amount whereas arginine and histidine were comparable to the *β*-galactosidase stable at moderate and low temperatures. Charged amino acids contribute to ion pair electrostatic interactions that are important binding force for maintaining conformational stability in surface of the proteins [41]. Helix region of *β*-galactosidase protein has the reverse order of charged amino acids; psychrophiles have higher content of these residues especially aspartic acid and glutamic acid. Lysine being an exception was higher in mesophiles and at both extreme ends its amount was low. In coil region charged amino acids were found statistically insignificant. This means that coil region of a protein is least involved in the temperature adaptation of proteins.

### 4.2. Sulphur Containing Amino Acids

Cysteine residues are known to play a dual role by both increasing thermostability by forming disulphide bridges and decreasing thermostability when available in free form as it is highly sensitive to oxidation at elevated temperature [42], though the present data on *β*-galactosidase showed that cysteine and methionine were statistically insignificant.

### 4.3. Tiny and Small Amino Acids

**Figure 5:**
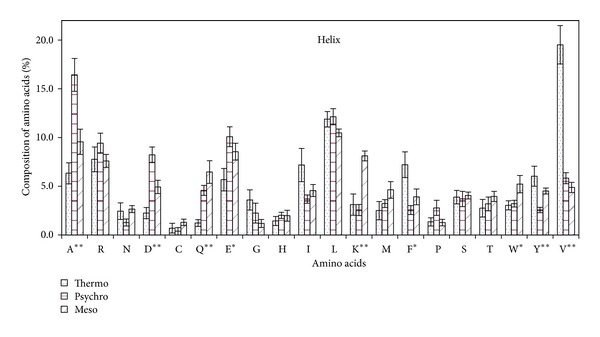
Percentage composition of amino acid in helix region of *β*-galactosidase from some thermophilic, psychrophilic, and mesophilic microorganisms.

**Figure 6:**
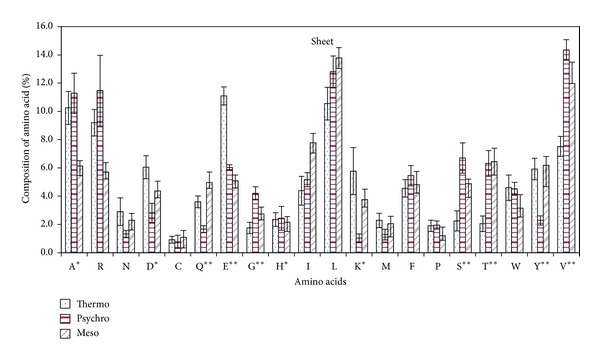
Percentage composition of amino acid in sheet region of *β*-galactosidase from some thermophilic, psychrophilic, and mesophilic microorganisms.

Among tiny amino acid residues, the relatively high percentage of alanine was noticeable in psychrophilic *β*-galactosidase and valine in thermophiles (Figure 1). Greater percentage of hydrophobic valine and isoleucine was expected in thermophilic proteins [30], as our data clearly showed high percentage of valine (Figure 1), especially helix region of thermophilic *β*-galactosidase (Figure 5). Higher percentage of valine in thermostable *β*-galactosidase is attributed to thermostabilization of protein. Alanine and valine being small nonpolar residue have been credited to be a good helix former [43] as the small side chain does not shield the backbone from solvent, allowing water to interact with the peptide carbonyl groups in a polyalanine helix. Compared to mesophiles and thermophiles, psychrophiles comprise a significantly higher proportion of amino acids that contribute to higher protein flexibility in the coil regions of proteins, such as those with tiny/small or neutral side chains. This explains the presence of high alanine content in coil region and high catalytic efficiency of psychrophilic *β*-galactosidase.

### 4.4. Glycine and Proline

The high percentage of glycine in coil region imparts flexibility to protein and being low in helix and sheet region observed accounts for the fact that it is not favored in these regions. Psychrophilic *β*-galactosidase has higher glycine in coil region which explains the high catalytic efficiency of cold active enzymes to some extent as it gives more flexibility aiding in its catalysis. Further structural analysis has also confirmed that their frequency is much greater in loops than helices [29, 44]. Helix region of thermostable *β*-galactosidase showed higher percentage of glycine as compared to mesophilic and psychrophilic *β*-galactosidase. Glycine is the smallest amino acid residue, devoid of side, and is flexible which aids in relaxation of steric hindrance of thermophilic enzymes and increases stability [42]. However, overall proteomes showed glycine to be less frequent in thermophiles [45], which supports our results (Figure 1).

Proline with pyrolidine ring structure has restricted conformations and was found to occur higher in thermophiles [44]. Higher proline percentage for thermostable protein shows its pronounced role in enhancing thermostability as it is more rigid than other amino acids and reduces the entropy of the main chain polypeptide decreasing the chance of unfolding at elevated temperatures [46]. The results in the present study have clearly showed that proline is higher in both ends of temperature when compared to moderate inhabitant.

### 4.5. Aromatic Amino Acids

Aromatic residues have been long assumed to lead to better thermostability of protein as weak polar interaction made by the aromatic ring of residues in phenylalanine is of enthalpic importance when compared to that of/with hydrogen bonding [39]. Aromatic residues can also be involved in interactions with nonprotein ligands that themselves contain aromatic groups via stacking interactions.

### 4.6. Hydrophobic Amino Acid

Both psychrophiles and thermophiles have higher content of hydrophobic amino acid residue. Isoleucine has a lot more bulkiness near the protein backbone and therefore preferred to lie within *β*-sheets. The present analysis has given a clear indication that isoleucine in helix region of thermophiles plays a significant role that is vital in the main stabilizing effects in proteins which leads to better packing of hydrophobic residues in helix region. Leucine being hydrophobic was found to be buried in protein hydrophobic cores which show a preference for being within alpha helices more so than in beta strands.

## 5. Conclusion

The protein sequence determines its structure which in turn ascertains its properties and function. During evolution of proteins they adapt to varying temperature by selecting the perfect combination of amino acid in their primary and secondary structure. The present investigation throws light on the preference and avoidance of amino acid in psychrophilic, mesophilic, and thermophilic *β*-galactosidase. This has strengthened our understanding of the temperature adaptation of proteins and results of the present study can be explored further to improve and design the properties of *β*-galactosidases for desired function.

## Figures and Tables

**Table 1:** Some *β*-galactosidase producing microorganisms and their optimum temperature for catalysis.

S/Number	Organism	UniProt Id	Temp. (°C)	pH	References
Thermophiles					
1	*Thermotoga maritima *	Q56307	85	6.5	[12]
2	*Sulfolobus solfataricus *	P22498	75	6.5	[11]
3	*Alicyclobacillus acidocaldarius *subsp. *acidocaldarius *	C8WV58	65	5.5	[9]
4	*Thermus* sp.	O54315	70	6.5	[13]
5	*Geobacillus kaustophilus *	P19668	70	7.0	[10, 14]
6	*Thermus brockianus *	Q9X6C6	70	7.0	[15]

Psychrophiles					
1	*Arthrobacter *sp.	Q44233	10	6.6	[16]
2	*Arthrobacter *sp. B7	Q59140	10	6.6	[17]
3	*Arthrobacter *sp.	C7ASJ5	10	7.2	[18]
4	*Arthrobacter psychrolactophilus *	Q08KN3	10	6.5	[7]
5	*Arthrobacter *sp. SB GN = lacZ	Q7WTU5	10	7.0	[19]
6	*Arthrobacter* sp. C2-2	Q8KRF6	10	8.0	[20, 21]

Mesophiles					
1	*Bacillus subtilis *(strain 168) O07012	O07012	30–40	6.5	[22]
2	*Escherichia coli *K12P00722	P00722	30	7.0	[23]
3	*Lactococcus lactis *subsp. * lactis *	Q48727	30	6.0	[24]
4	*Yersinia pestis *	Q7CIZ3	30	6.5	[25]
5	*Bacillus licheniformis * ATCC14580	Q65CX4	30	6.5	[26]
6	*Lactobacillus delbrueckii *subsp.* bulgaricus *	P0C1Y0	30	6.0	[27]
